# “Calming the nightmares”: A qualitative study of a socially assistive robot for sensory and emotional support in individuals with eating disorders and PTSD

**DOI:** 10.1371/journal.pone.0325469

**Published:** 2025-06-06

**Authors:** Dimitri Chubinidze, Zhuo Li, Philippa Croft, Brittany Nodding, Petr Slovak, Kate Tchanturia

**Affiliations:** 1 Department of Psychological Medicine, Institute of Psychiatry, Psychology and Neuroscience (IoPPN), King’s College London, London, United Kingdom; 2 National Eating Disorders Service, South London and Maudsley NHS Foundation Trust, London, United Kingdom; 3 Department of Informatics, King’s College London, London, United Kingdom; 4 Department of Psychology, Ilia State University, Tbilisi, Georgia; Sapienza University of Rome: Universita degli Studi di Roma La Sapienza, ITALY

## Abstract

Individuals with eating disorders (ED) and co-occurring post-traumatic stress disorder (PTSD) often face difficulties with sensory overload and emotion regulation (ER), which can make treatment more complex. Assistive devices that offer real-time support are needed to enhance therapeutic interventions. This qualitative pilot study explored the engagement, acceptability, and perceived impact of Purrble, a socially assistive robot, as an adjunct tool for sensory and ER support. Nine participants (8 female, 1 non-binary, aged 20–55) were recruited from an ED service and engaged with Purrble over a 10-day period following a sensory well-being workshop. Participants recorded their use of Purrble and daily reflections in diaries and participated in a focus group. Thematic analysis revealed three themes: (1) Integration into daily life, where participants highlighted Purrble’s portability and ease of use in managing anxiety across various settings; (2) Managing PTSD-related difficulties, such as calming nightmares, reducing sensory overload, and providing emotional comfort; and (3) Challenges and improvements, where participants suggested modifications, such as adding a night mode and better sound control. This study provides preliminary evidence that an assistive device can support individuals with ED and PTSD, particularly in managing sleep disturbances, overstimulation, and feelings of emotional isolation. Further research using standardised methodologies is needed to expand on these preliminary findings.

## Introduction

Eating Disorders (ED) are complex conditions that affect psychological and physical well-being [1,2]. ED are often linked to factors such as adverse childhood experiences (ACEs) and trauma, which play an important role in the development and maintenance of the disorder [3,4]. Rienecke and colleagues found that adults with ED report higher rates of ACEs compared to the general population, emphasising the lasting impact of childhood trauma on ED [5]. Trauma, including physical and emotional abuse, are particularly common among individuals with anorexia nervosa (AN) and bulimia nervosa (BN) [6]. Tagay and co-authors reported that 23.1% of AN and 25.5% of BN patients meet the diagnostic criteria for PTSD, with trauma exposure worsening ED symptoms [7]. The co-occurrence of trauma and disordered eating complicates treatment, as their interaction creates additional challenges during recovery [8–12].

Those with comorbid ED and PTSD tend to experience more severe ED symptoms, emotional distress, and ER difficulties than those with ED alone [13–16]. These difficulties are linked to shared maladaptive regulation strategies such as avoidance and suppression, often rooted in early trauma [17]. A recent meta-analysis identified rumination and non-acceptance of emotions as key difficulties in ED, pointing to the value of approaches that support acceptance, reappraisal, and emotional awareness [18]. Ecological momentary assessment studies show that comorbid PTSD is linked to more intense and rapid emotional shifts [19]. These emotional fluctuations, along with hyperarousal and dissociation, may hinder the use of cognitive strategies like reappraisal in overstimulating contexts. Individuals with ED often report heightened sensitivity to food-related sensations, textures, or smells, which can trigger emotional responses and reinforce disordered eating behaviours [20–23]. Similarly, PTSD is linked to increased reactivity to auditory, tactile, and visual stimuli, leading to hyperarousal and distress in everyday situations [24–27]. These sensory sensitivities are closely linked with emotional dysregulation, suggesting that both may serve as critical intervention targets across diagnoses.

While cognitive, dialectical behavioural, and acceptance-based therapies offer benefits [28], their effectiveness can be modest and inconsistent. These treatments are based on cognitive processes and verbal communication, which may not be accessible during states of emotional or sensory overwhelm. To address this gap, recent reviews highlight digital tools—like mobile apps, wearables, and immersive technologies—as a promising adjunct for ER [29]. Digital ER is an emerging field that brings psychological theory into dialogue with interactive technology, supporting real-time strategies like attentional deployment and response modulation in more context-sensitive ways [30]. Enhancement of therapeutic practice through interactive technology opens new possibilities and socially assistive tools such as therapeutic devices may offer support where conventional methods are limited. Therefore, there is a growing interest in exploring scalable, real-time interventions that support sensory and ER in an accessible way.

### Purrble – an adjunct intervention for sensory and ER

To improve treatment outcomes, there has been increasing interest in incorporating adjunct therapeutic interventions that specifically address sensory overload and ER. Integrating innovative, evidence-based approaches alongside traditional talk therapies has shown promise [31]. The PEACE (Pathway for Eating Disorders and Autism developed from Clinical Experience) care pathway, reflects this shift, having introduced sensory-informed resources and environmental adaptations that help patients, especially those with neurodivergent profiles, build personalised regulation strategies [32–35]. These developments include psychoeducational resources and well-being workshops, which support patients to create personalised sensory toolkits. Such interventions have proven effective in managing sensory and emotional challenges [36,37]. Although particularly relevant for individuals with neurodivergent conditions and ED, these adjunct interventions can also benefit neurotypical ED patients.

Alongside these approaches, technology-enhanced interventions are gaining attention. One such intervention is Purrble, a socially assistive device designed to provide real-time support for sensory overload and ER. Grounded in Gross’s extended process model of ER [38], Purrble specifically targets two regulatory mechanisms: attentional deployment – by shifting focus away from distressing stimuli – and response modulation – by providing calming, tactile feedback that supports downregulation of physiological arousal. These mechanisms are supported by sensory features such as soft textures, rhythmic vibrations, and simulated heartbeat, which draw on principles from sensory integration and human-animal interaction [39].

In this context, Purrble can complement cognitively demanding strategies, which may be difficult to access during states of distress. Prior studies have shown its acceptability and potential therapeutic impact across diverse populations, including children [40–42], highly anxious students [43], and individuals with ED [44]. Its interactive design encourages repeated use, affective engagement, and gradual change in self-regulation habits.

Building on earlier feasibility work [44], this study aims to explore the engagement, acceptability, and perceived impact of an assistive device for real-time support in managing sensory systems and ER challenges in individuals with ED and comorbid PTSD. Understanding the potential of such tools is essential for developing trauma-informed interventions that can support recovery in individuals affected by both conditions.

## Materials and methods

### Research design

This study employed a qualitative approach, using thematic analysis [45] to explore participants’ experiences with Purrble. The methodology aligns with the aim of capturing participants’ lived experiences and subjective perceptions of the device. The study adheres to the Standards for Reporting Qualitative Research (SRQR) [46].

### Intervention

**Fig 1:**
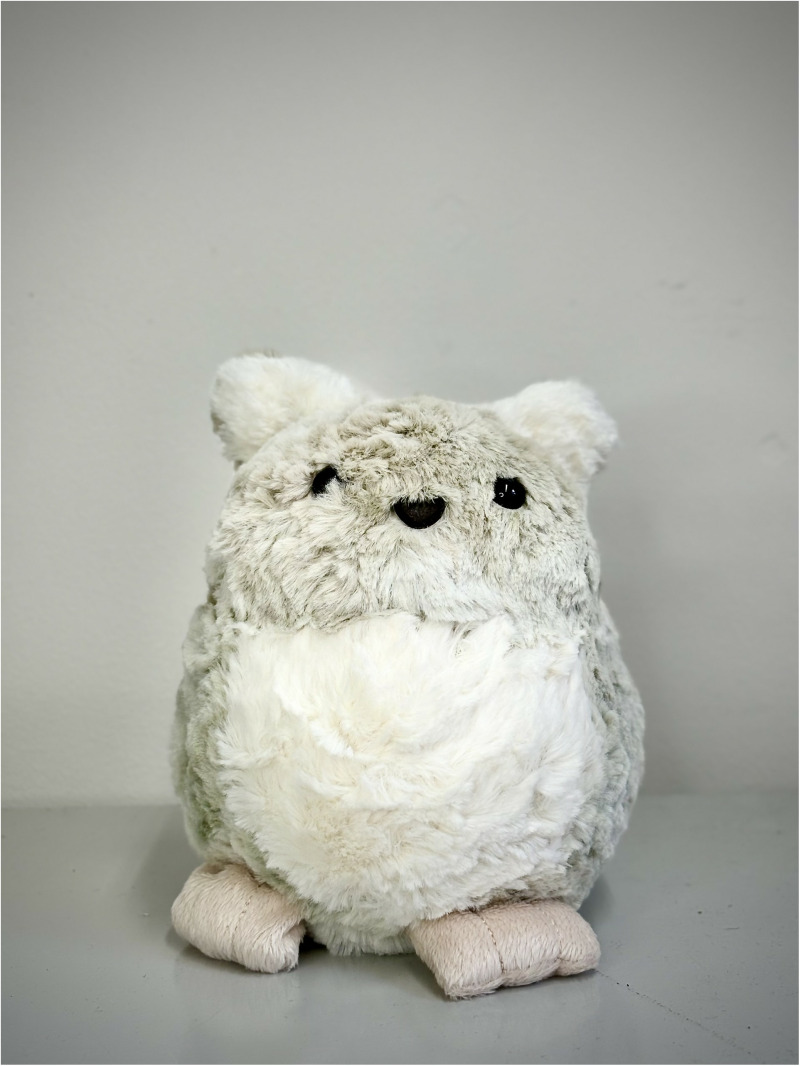
Purrble.

Purrble is a socially assistive device designed as a soft, interactive plush toy with built-in sensors and haptic feedback that simulates heartbeat [47] (Fig 1). Each interaction begins with the toy in an “agitated” state, vibrating with a rapid heartbeat that slows gradually in response to calm, sustained stroking. After about a minute of tactile engagement, it transitions into a gentle purring state. This brief interaction loop is guided by the user’s touch and can offer emotional support through sensory engagement and co-regulation.

### Ethics, sampling, and procedure

The study was approved by the South London and Maudsley Psychological Medicine & Older Adults Operational Directorate (MHOA&D and PMIC CAGs) (335).

Purposive sampling was used to recruit participants from a specialist ED outpatient service. The sample size was deemed appropriate considering the exploratory nature of this qualitative feasibility study, which focused on usability and user experience rather than thematic saturation, and was informed by prior research employing similar methods [44].

Inclusion criteria included a confirmed ED diagnosis and a formal or suspected PTSD diagnosis, both established through routine multidisciplinary clinical assessments in accordance with DSM-5 criteria. Exclusion criteria were severe cognitive impairment or acute psychiatric instability that could hinder participation.

The study was conducted between April and June, 2024, and carried out in three phases:

Phase 1: Sensory well-being workshop. A one-hour, in-person session, which provided psychoeducation on sensory regulation and included a hands on activity to create a personalized sensory item (e.g., scented cream, glitter jar. details of the workshops [36,37]). At the end, Purrble was introduced and distributed with brief verbal and written instructions to support spontaneous, in-situ use.

Phase 2: 10-day use of Purrble. Participants were asked to use the device as needed over a 10-day period. They completed a structured daily diary including a 10-point visual analogue scale (VAS) to rate emotional and sensory states, contextual prompts (e.g., where, when, why Purrble was used), and open-ended reflection fields. The diary format followed a previous study [44].

Phase 3: Focus group. Following the 10-day interaction period, participants attended a 45-minute, in-person focus group facilitated by DC and PC. A semi-structured guide was used, and sessions were audio-recorded and transcribed for analysis.

### Analysis

Qualitative data regarding sensory and emotional processing were collected through participant diaries and focus group discussions. The data were transcribed verbatim and analysed using thematic analysis, following Braun and Clarke’s six-phase framework [45]. Transcripts were imported into NVivo 14 to facilitate data management and coding. Initial coding was performed by DC and subsequently reviewed in collaboration with ZL, KT, and BN. Any discrepancies in coding were discussed and resolved through consensus. Quantitative VAS data were visualised to track usage trends and perceived emotional and sensory changes.

Themes, along with anonymised participant quotes (e.g., P1, P2), are presented in Section 3. Focus group quotes are labeled ‘FG’. The qualitative and quantitative findings are presented together under relevant themes, highlighting usage patterns of the device and its perceived impact on ER and sensory sensitivity.

### Diagnostic confirmation

ED diagnoses were made using DSM-5 criteria by consultant psychiatrists. PTSD status was determined by the clinical team based on clinical and risk assessments, and consensus during routine multidisciplinary meetings.

## Results

Nine adult participants completed the full study. Two workshops were held, each with 4–6 participants. One participant was excluded for incomplete diary and non-attendance at the focus group.

Table 1 summarises the participant demographics. Most participants were female (n = 8), with one identifying as non-binary. The average age was 30 years (range: 20–55). The majority were White British (n = 5, other ethnic backgrounds (n = 4).

**Table 1:** Summary of participants’ health and demographic characteristics.

Variables	*n* = 9
Age, mean (SD)	30.0 (10.19)
Gender, female *n* (%)	8 (88.89%)
Ethnicity, *n* (%)	
White British	5 (55.56%)
White Other	1 (11.11%)
Black British	1 (11.11%)
Black African	1 (11.11%)
Mixed	1 (11.11%)
ED Diagnosis, *n* (%)	
AN restrictive subtype	5 (55.56%)
AN binge-purge subtype	1 (11.11%)
Bulimia Nervosa	3 (33.33%)
Duration of ED in years, mean (SD)	13.83 (13.25)
Missing, *n* (%)	4 (19%)
BMI on admission, mean (SD)	24.34 (13.35)
Comorbidity, *n* (%)	
PTSD (including diagnosis and traits)	9 (100%)
MDD	1 (11.11%)
Anxiety	2 (22.22%)
OCD	1 (11.11%)

Abbreviations: AN—Anorexia Nervosa; ED—Eating Disorder; BMI—Body Mass Index; OCD—Obsessive Compulsive Disorder; MDD—Major Depressive Disorder; N—Number of Participants; SD – Standard Deviation

**Fig 2:**
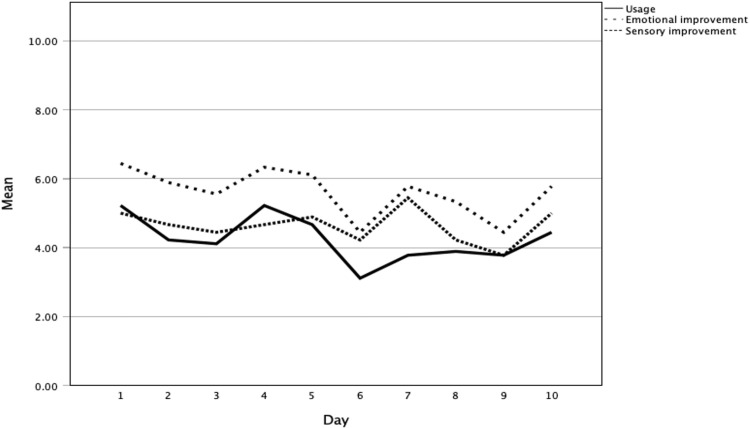
Trends in Purrble usage, emotional, and sensory improvements over 10 days.

Participants engaged with Purrble approximately 42 times over 10 days, according to diary data. Fig 2 illustrates patterns of use, along with changes in ER and sensory sensitivity. Improvements in participants’ emotional state and sensory sensitivities followed a similar pattern to their engagement with Purrble.

Qualitative analysis yielded four primary themes, each reflecting both benefits and limitations of Purrble’s use: (1) Integration of Purrble into daily life, (2) Managing PTSD-Related dysregulation: sleep, sensory overload, and emotional isolation, (3) Tactile comfort and grounding mechanism, (4) Suggested improvements for device usability. A thematic summary of these categories, including illustrative quotes, is presented in Table 2. The themes described below reflect participants’ lived experiences and directly address our research questions around engagement, emotional/sensory regulation, and acceptability of the Purrble device.

**Table 2:** Thematic summary with illustrative quotes.

Theme	Subdomain	Illustrative Quotes
Integration into Daily Life	Mobility, context-based use	“I used it on the train… it calmed me down” “I carried it to the kitchen”
Managing PTSD-related Dysregulation	Sleep support, overstimulation, emotional isolation	“It calmed nightmares” “Helped me cope with slamming doors” “It helped me feel less alone.”
Tactile Comfort and Grounding	Soothing touch, breathing regulation	“I matched my breathing to its heartbeat” “Stroking it was calming.”
Suggested Improvements	Practical issues, sensory mismatch	“The fast heartbeat made me anxious” “My puppy tried to eat it.”

*This table complements the qualitative themes and supports a nuanced understanding of Purrble’s acceptability, emotional impact, and sensory affordances. The themes captured a balanced picture of Purrble’s use – participants reported meaningful emotional and sensory benefits, while also identifying usability challenges and design limitations that shaped their experiences.*

### Integration of Purrble into daily life

#### Mobility and use outside the home.

Participants described Purrble as portable and easy to use daily, at home or outside. Several participants highlighted its benefits whilst travelling, particularly in managing anxiety in public spaces. For example, one participant explained how she frequently *“took Purrble on the train... because I really hate the train, and it calmed me down”* [FG1], while another noted its utility during air travel, sharing, *“I traveled, so I took it on the airplane with me. I felt like I used it more when I was away from home.”* [FG2].

In home environments, Purrble was integrated into various spaces and activities, for example: *“I used my Purrble in the living room a lot because I usually chill there. Sometimes in my bed and in the kitchen”* [FG2], while another participant mentioned, *“I carried it to the kitchen as well. I would hold it, or if I was cooking and waiting for something to finish, I would sit with it”* [FG2]. This adaptability suggests that Purrble was easy to use at home.

Some participants described using the device outside, for example: *“I also took Purrble to therapy this week. It was helpful to have something else to focus on, like a fidget toy, rather than just my own thoughts”* [FG1], or: *“I used it all day, every day. Even when I was at work, it stayed in my bag. I’ve just had an appointment with the dietitian, and it’s still in my bag. I’ve taken it everywhere”* [FG2].

#### Targeted interaction and relationship dynamics.

Over time, participants reported changes in how they used Purrble, with some initially engaging with the device more frequently but later adopting more targeted use based on specific emotional needs. One participant explained, *“I initially used it for longer periods at the beginning, so I was using it like seven to eight times a day... Then towards the end, I used it more when I felt like I needed it”* [FG2].

Some participants expressed initial skepticism about using Purrble, particularly due to its toy-like appearance, but these feelings often changed after experiencing its benefits. One participant reported, *“I was kind of skeptical at first. I’ve never been one for ‘toys,’ even as a kid, so it was a bit of a leap for me to consider it. Actually, though, after using it the first time and finding it helpful, it was something that I actively reached for”* [FG1]. Similarly, another participant shared how she initially felt self-conscious about using Purrble around family members but eventually embraced it, stating, *“Initially, I was going to keep it a secret from my husband because I felt a bit silly using a toy. But then I thought I wouldn’t get the full use out of it if I hid it away. My husband said, ‘It’s fine, whatever helps you.’ My family thinks it’s quite nice to see me sitting with it”* [FG1].

This evolving relationship with the device suggests that, despite initial hesitation, participants came to view Purrble as a useful and reliable tool. One participant confidently concluded, *“I definitely will use Purrble in the future, 100%. I really like stuff like this, and I think it’s helped, so I will keep using it”* [FG2].

### Managing PTSD-related dysregulation: sleep, sensory overload, and emotional isolation

#### Sleep regulation and support.

Participants frequently discussed using Purrble to manage distress caused by nightmares and night terrors, which are common symptoms of PTSD. The device was often described as a grounding tool, aiding emotional regulation during nighttime episodes of hyperarousal and dissociation.

One participant notably described Purrble as “calming the nightmares,” highlighting its role in providing immediate support and grounding. Others reported that Purrble helped them regain composure after waking from nightmares. As one participant described*, “I often am quite distressed and find it hard to ground myself after nightmares, but having Purrble there, regulating my breathing alongside it’s comforting presence, really helped. The vibration, in particular, helped provide that connection to the ‘here and now’”* [FG1]. Another participant shared a similar experience, stating, *“It was particularly helpful when I woke up from a night terror and felt confused and dissociated”* [P2]. These accounts suggest that Purrble provided an immediate, calming effect, helping participants ground themselves during moments of distress.

In addition to supporting participants after nightmares, Purrble was also used as a sleep aid. Some participants, particularly those who experienced heightened anxiety or racing thoughts at night, found that holding Purrble made it easier to relax and fall asleep. One participant explained, *“I slept with Purrble every night as my mind goes into overdrive at night, and I find it extremely hard to sleep. It has really helped me with that, and I’ve been able to go to sleep much easier than before”* [P5]. Others echoed similar experiences, emphasizing Purrble’s dual role in helping them fall asleep and manage anxiety upon waking from nightmares*: “It was particularly helpful at night, both in falling asleep and after nightmares”* [P4].

For some participants, Purrble became a key strategy for managing trauma-related sleep disturbances. One participant reflected, *“I think I have discovered a new coping strategy for trauma nightmares”* [P2]. This suggests that participants began to view Purrble as a useful tool for addressing the specific challenges associated with PTSD-related sleep issues.

#### Managing overstimulation.

Participants found Purrble effective in managing sensory overload, a common experience among individuals with PTSD and ED. The device supported regulation of responses to environmental stimuli, helping to reduce the impact of triggers such as loud noises or sudden movements, which might otherwise intensify stress and anxiety.

One participant shared, *“ Purrble helped me handle feeling overwhelmed by things around me. I became less bothered by small sounds or movements and could better cope with different sensations”* [FG1]. Similarly, another noted how it was useful in moments of sudden overstimulation, stating, *“I used it when sudden noises would set me off, like my housemate slamming a door, and it helped me calm down before I became overwhelmed”* [FG2].

Purrble was described as an effective tool for preventing anxiety escalation during episodes of sensory overload. One participant explained, *“When I got overstimulated, Purrble was an effective way to soothe myself before my anxiety spiraled out of control”* [P9].

In addition to its role in managing acute sensory triggers, participants indicated that regular use of Purrble increased their tolerance to sensory input over time. One participant noted, *“I found that using Purrble made me less likely to be tipped over the edge by minor sensory inputs. It was like my ability to tolerate sensory inputs increased”* [FG1].

#### Coping with emotional isolation and loneliness.

Participants described Purrble as a comforting presence that helped them cope with feelings of loneliness and emotional isolation, common experiences for individuals with PTSD and EDs. Purrble provided emotional support by offering a sense of companionship, which helped mitigate feelings of sadness and depressive symptoms.

One participant explained, *“I used Purrble more for sadness and when I felt alone, and it really helped me feel less isolated”* [FG1]. Others emphasized how Purrble offered reassurance even when it wasn’t actively held. As one participant noted*, “It would sit with me at meals or while I worked, and just having it there was comforting’* [FG1].”

Some participants highlighted Purrble’s role specifically in lifting their mood during difficult emotional moments. One stated, *“I used it when I was feeling lonely and down, and it helped lift my mood”* [P1]. Another described using Purrble more as a companion throughout the day, sharing, *“I wouldn’t always hold it, but having it near me as a visual reminder was reassuring.”* [FG1].

Overall, Purrble provided participants with emotional comfort and a sense of connection, helping reduce feelings of loneliness and offering support during moments of isolation and low mood.

### Tactile comfort and grounding

Participants frequently described Purrble as a source of physical comfort, which was particularly valuable during moments of anxiety, sadness, or emotional distress. The tactile experience of holding Purrble, especially its heartbeat feature, was often reported as calming influence that helped participants regulate their emotions and feel grounded.

One participant said, *“The heartbeat sensation was soothing, and I matched my breathing to it, which calmed me down”* [P2]. Similarly, another participant noted, “Stroking Purrble when I was stressed at work was immediately calming and stopped my anxiety from escalating” [FG1]. Purrble’s tactile features, such as its soft fur and rhythmic purring, provided comfort that participants described as particularly effective when other sources of support, like pets, were unavailable: *“Cuddling Purrble when I needed physical comfort, especially when my pets weren’t around, was incredibly soothing”* [P2].

In moments of heightened distress, Purrble functioned as an emotional grounding tool, helping participants shift focus away from overwhelming emotions. One participant explained, *“I found Purrble helpful in moments of high stress. I usually want to destroy things when I’m stressed... but with Purrble, it helped me focus on something else”* [FG1].

Purrble’s presence provided not only tactile comfort but also a sense of stability during stressful situations. One participant described how *“the physical sensation of the purring and vibration was calming, and I found it helpful to match my breathing to the pace of Purrble’s heartbeat to eventually slow my breathing down”* [P2]. This grounding effect was particularly useful during moments of high anxiety or emotional overwhelm: *“I found Purrble so comforting and helpful. It gave me a focus and helped to ground me in times of high stress”* [P3].

### Suggested improvements for device

While participants generally found Purrble helpful, several suggestions were made to improve the device. Some users reported that certain features, such as the faster heartbeat and specific sounds, occasionally increased their anxiety rather than providing comfort. One participant noted, *“I did find the faster heart rates and squeaks a bit anxiety-provoking”* [FG1].

Practical difficulties were also noted, especially for those with pets. One participant shared, *“I didn’t have much consistency in using it because my puppy wanted to eat it – got jealous”* [FG1].

A common request to improve the device was the addition of a night mode or sleep mode that would turn off the device after a set period, making it more suitable for night-time use. One participant shared, *“A night mode that stops after a few minutes would be a game-changer”* [FG2].

Adjustments to sound features were also suggested, such as the option to turn off or control the volume of the device. One participant mentioned, *“The noises need to be a bit louder because they’re hard to hear unless pressed against the ear”* [P5], while another recommended shortening the purring duration, *“It goes on for too long and isn’t as soothing”* [FG2].

## Discussion

This exploratory study builds on previous research examining the use of socially assistive devices in diverse ED clinical settings [44]. In the present study, we explored the feasibility of the Purrble – in a different clinical context, focusing on individuals with ED and comorbid PTSD. Our aim was to assess engagement with the device, its acceptability, and participants’ perceived impact of its potential to support sensory overload and ER. While the findings are preliminary, they align with earlier work and suggest that participants engaged with Purrble in ways they found personally helpful. These findings add to the growing evidence base on socially assistive technologies for individuals with ED and complex comorbidities.

### Engagement and acceptability

Participants’ self-reported engagement rates with Purrble showed high levels of interaction, particularly during the initial phase of the study. As the observational period progressed, participants shifted from frequent exploratory use to more targeted, situational use. This suggests that it became a personalised tool, employed specifically during moments of heightened emotional or sensory overwhelm. This pattern of adaptive use is consistent with previous research [43,44], highlighting its role in providing real-time support. Interestingly, this sustained engagement contrasts with broader trends in digital interventions, where engagement typically declines significantly over time [48,49]. Ongoing engagement remains a key challenge in traditional therapeutic approaches [50], highlighting Purrble’s potential to address this gap and offer a more consistent form of support.

Furthermore, Purrble’s integration into daily routines—whether at home, during travel, or in therapeutic settings—demonstrates the flexibility and added benefit. These findings align with existing literature showing that portable, real-time interventions and monitoring are effective in supporting individuals with trauma experiences, aiding in the management of both emotional and sensory dysregulation [51,52]. However, unlike some digital tools that rely on screen-based or verbal input, Purrble’s embodied, tactile interface may offer unique advantages in moments of emotional overwhelm—particularly for those with difficulties in verbal processing or sensory sensitivity. Prior studies have noted the limited impact of verbal-only or app-based tools under high arousal conditions [30], suggesting a need for tools like Purrble that provide multi-sensory, non-verbal interaction. This positions our findings as a meaningful extension to the literature by addressing these gaps through an embodied, co-regulatory mechanism.

### Perceived impact on PTSD complications

#### Nightmares and sleep disturbances.

Participants frequently used Purrble to manage nightmares and sleep disturbances, common symptoms of PTSD [53,54]. Although there is growing interest in digital interventions for nightmares, particularly in the realm of biofeedback, research on their efficacy remains limited [55]. For instance, smart watch haptic feedback interventions show potential in predicting and preventing nightmares through tactile reminders [56], but support options for post-nightmare recovery or disturbed sleep are scarce. In this context, Purrble’s tactile feedback, provided by its heartbeat feature, proved to be a valuable support for participants emotionally ground themselves after nightmares. By synchronising their breathing with the device’s heartbeat, participants experienced a calming effect that aided emotional regulation, as documented in previous studies [41,42,44]. Furthermore, our results showed that Purrble’s features helped reduce hyperarousal and allowed participants to regain control during episodes of dissociation or confusion upon waking.

#### Sensory overload.

A frequent challenge for individuals with EDs and PTSD, was another area where Purrble provided support. Participants described the device as an effective tool for managing heightened sensitivity to auditory, visual, and tactile stimuli. By offering an immediate sensory input, Purrble helped participants cope with overwhelming environmental triggers, preventing anxiety from escalating and allowing them to better manage their emotional responses. This reinforces existing evidence of Purrble as a promising tool for sensory regulation [44].

#### Isolation and companionship.

Participants consistently reported that Purrble provided emotional comfort and support, helping to alleviate feelings of loneliness and emotional isolation, both of which are common in PTSD and ED populations [57–60]. Purrble’s presence, even when not actively held, was described as reassuring and supportive, suggesting that its role extended beyond tactile stimulation to offering psychological comfort through its presence. This finding aligns with existing literature on the potential of socially assistive technologies to enhance emotional connection [61], highlighting Purrble’s as a companion for individuals experiencing social isolation as part of their PTSD or ED treatment journey.

### Usability challenges and suggested improvements

Some participants offered suggestions for more customisable options, such as a night mode for better usability during sleep, volume control, or shortening the duration. These findings align with a study with individuals with ED, where similar suggestions for device improvements were suggested [44]. Participants’ views could be translated into personalised improvements of this device.

The themes identified in this study reflect the two ER mechanisms targeted by the intervention: attentional deployment and response modulation [38,39]. For instance, participants described using Purrble to shift focus away from distressing thoughts or external triggers (attentional deployment), particularly during moments of sensory overload or emotional isolation. Simultaneously, the device’s tactile interaction and calming sensory feedback supported physiological downregulation and emotional grounding (response modulation), as seen in managing sleep disturbances and high arousal states. These findings suggest that participants were intuitively engaging with the device in ways consistent with its theoretical design.

### Clinical implications and limitations

This pilot study suggests that Purrble may serve as a valuable adjunct to existing interventions targeting emotional and sensory regulation in individuals with EDs and comorbid PTSD. Its real-time support and adaptability align with the growing emphasis on personalised, technology-enhanced mental health interventions. By providing immediate emotional grounding and sensory management, Purrble demonstrated the potential to address critical needs such as mitigating sensory overload, alleviating emotional isolation, and managing PTSD-related challenges, including sleep disturbances.

Many participants described Purrble as a source of emotional connection, helping to reduce feelings of loneliness and detachment prevalent in EDs and PTSD. Clinicians can use this to foster a sense of support for patients struggling with emotional detachment.

In addition, difficulties with emotion regulation are common among individuals with EDs; therefore, Purrble may benefit patients beyond those with PTSD and can be used in conjunction with other therapies that focus on emotional regulation. Improved emotional regulation during therapy sessions can enhance patient engagement, potentially leading to better treatment outcomes. Unlike many digital interventions that lose user engagement over time, Purrble demonstrated consistent use due to its ease of integration into daily routines. This highlights its potential for long-term application in patient care plans. Clinicians can encourage patients to incorporate Purrble into their daily habits, such as during mealtimes, commutes, therapy sessions, or bedtime routines. It may also be helpful in waiting rooms before appointments, as a grounding tool during group sessions, or as a transitional object during periods of increased distress, such as during mealtimes, stressful moments, or urges to binge or purge, to support ongoing emotional regulation.

In more intensive treatment settings, Purrble can be utilized for meal support, addressing the emotional dysregulation that often occurs around meals and eating. In group treatments, Purrble may serve as an effective tool to foster engagement, particularly for individuals who feel uncomfortable with active participation. For virtual sessions, Purrble can act as a tangible, supportive tool both during and between therapy sessions, contributing to continuity of care. Upon discharge from treatment settings, Purrble can be provided as a take-home resource to support ongoing recovery and reduce the risk of relapse.

By supporting both the sensory overload and ER needs of individuals with ED, Purrble can be integrated into various stages of the treatment process, from acute intervention to long-term maintenance. The results of the Purrble evaluation studies in clinical settings highlights the potential for developing other socially assistive devices for clinical populations.

Clinicians should keep in mind that, while Purrble may benefit many patients, it may not be suitable for everyone. Its use should be approached collaboratively with each patient to determine whether it can support their treatment effectively.

Despite these promising outcomes, several limitations must be acknowledged. This was a small-scale qualitative feasibility study designed to explore user experiences, not to test intervention efficacy or compare outcomes. The small sample size limits generalizability of the findings to broader clinical populations. The reliance on self-reported data introduces the potential for response bias, including social desirability or recall effects. These methodological limitations highlight the need for future research employing larger, controlled trials with mixed approaches to validate and expand upon the present findings.

## Conclusion

This pilot study demonstrates that Purrble is a promising adjunct tool for addressing sensory overload and ER challenges in individuals with ED and comorbid PTSD. Participants reported its benefits in managing distress, particularly during sleep disturbances, sensory overload, and emotional isolation. These findings highlight Purrble’s potential to provide real-time, portable support in trauma-informed care settings.

Additionally, the study reveals areas for device improvement, such as the need for enhanced sensory features and greater customisation. These refinements could further enhance usability of the device.

This study contributes to the growing evidence on socially assistive devices, highlighting their potential applications in populations with complex comorbidities. The findings suggest these devices could serve as adjuncts to talking therapies, offering immediate and practical support for emotion and sensory regulation.

Future research should focus on replicating these preliminary findings through controlled trials and standardized methodologies, exploring both clinical outcomes and long-term engagement. Additional studies could examine Purrble’s effectiveness in other diagnostic groups—such as general anxiety disorders, mood disorders, neurodevelopmental conditions – and assess its integration into diverse treatment settings, including inpatient care, community-based programs, and telehealth environments. These efforts will be essential to evaluate the long-term therapeutic potential of the device and determine its broader clinical applicability.
